# Preparation and Characterization of Perfluoropolyether-Silane@Ethye Cellulose Polymeric Microcapsules

**DOI:** 10.3390/polym16020169

**Published:** 2024-01-05

**Authors:** Zijian Song, Ruijie Chen, Zilang Huang, Yucheng Gong, Haitao Zhao

**Affiliations:** 1 College of Civil and Transportation Engineering, Hohai University, 1# Xikang Road, Nanjing 210098, China; 2 College of Mechanics and Materials, Hohai University, 8# West Focheng Road, Nanjing 210098, China

**Keywords:** PFPE-silane, EC, microcapsule, hydrophobicity

## Abstract

A novel polymeric microcapsule was designed and synthesized using perfluoropolyether silane (PFPE-silane) as a superhydrophobic core material and ethyl cellulose (EC) as a shell material. The effects of the stirring rate and the core-to-shell ratio on the synthesized microcapsules were investigated. The physicochemical properties of the polymeric microcapsules were evaluated using scanning electron microscopy, fourier transform infrared spectroscopy, thermogravimetric analysis, laser particle size analysis, and wettability analysis. The results showed that when the stirring rate was 650 rpm and the core-to-shell ratio was 1:1, well-distributed and uniformly dispersed microcapsules could be obtained. The results also indicated that the prepared polymeric microcapsules were spherical particles with micropores on the surface, and they had an average particle size of 165.71 μm. The EC shells could effectively prevent the thermal decomposition of PFPE-silane during cement hydration, and the PFPE-silane also exhibited excellent hydrophobicity. The specially designed structure of this polymeric microcapsule suggests its potential for enhancing the corrosion resistance of reinforced concrete structures.

## 1. Introduction

Reinforced concrete has been widely used in civil engineering projects due to its complementary advantages. However, the porous structure and hydrophilicity of cementitious materials allow water to enter the material through pores and cracks. The aggressive ions in the water can have adverse effects on the cementitious materials, leading to various performance deteriorations such as rebar corrosion and carbonization [1,2]. This irreversible damage necessitates the urgent study of effective methods to slow down the water penetration rate.

Currently, there have been many reports on the hydrophobic modification of cementitious materials [3,4]. These reports explored the use of surface and integral hydrophobicity methods to improve the hydrophobicity of cementitious materials by incorporating hydrophobicity additives [5,6,7]. However, these approaches inevitably resulted in the loss of the compressive strength of cementitious materials. To address this issue, the use of hydrophobic compounds wrapped with polymeric microcapsules has been proposed. The microencapsulation technique can improve the hydrophobic properties without significantly weakening the compressive strength of cementitious materials. By triggering the capsules and releasing their core content, cracks can be effectively sealed, the invasion of erosive media can be impeded, and the corrosion of reinforced concrete can be mitigated. Commonly used polymeric microcapsules in concrete include urea-formaldehyde resin (UF) microcapsules [8], polyurea microcapsules [9], and ethyl cellulose (EC) microcapsules [10]. Among these, EC microcapsules stand out for their encapsulation properties in multiple functional substances, the absence of toxic release in service, and high preparation stability.

With the advancement of the chemical industry, various methods have been developed for preparing microcapsules. One commonly used method is the spray drying method [11], which involves dispersing a solution of core material and wall material into small droplets using a sprayer. These droplets are then exposed to hot air (or cold air) to evaporate the solvent, resulting in a dry powder product. This method offers the advantages of a short processing time and a fast solvent drying rate. However, it has some drawbacks, such as the need for large equipment, high energy consumption, and high costs. Another method, known as interfacial polymerization, involves the reaction of different compounds at the interface to produce microcapsules. Unfortunately, this method often leads to incomplete reactions and the presence of impurities in the final product. In contrast, emulsion solvent evaporation [12,13] is a popular choice due to its simplicity, short processing time, and the absence of chemical reactions. This method involves four main steps: dissolving the core material and wall material, preparing a stable emulsion, evaporating the solvent, and finally washing and drying the microcapsules. Therefore, for this experiment, emulsion solvent evaporation was selected as the preferred technique for microcapsule preparation. PFPE is a highly effective hydrophobic agent that can create a hydrophobic layer on material surfaces due to its hydrophobicity and oleophobicity. This makes it a popular choice for anti-fingerprint coatings in the smartphone industry [14,15,16]. PFPE-based molecules typically consist of PFPE chains and functional end groups. The siloxane end in PFPE-silane can form -Si-O- bonds with the material substrate through hydrolysis and condensation reactions, which enhances the adhesion between hydrophobic coatings and substrates [17,18,19]. PFPE segments tend to form self-assembled monolayers (SAMs) on the material surface, imparting excellent hydrophobicity, repulsion, and fingerprint resistance [20,21]. There is a considerable body of literature exploring the properties of PFPE and its modified compounds on glass surfaces. Yong Nam Ahn et al. [22] conducted a comprehensive study on the adsorption characteristics of PFPE-silane molecules on the surface of hydroxylated glass silica at the atomic level. Their findings revealed that despite having three silanol branches at the molecular end, PFPE-silane predominantly forms a single siloxane bond on the surface. Additionally, the study determined that PFPE-silane molecules adsorb parallel to the surface, inhibiting the formation of intermolecular cross-linking structures and resulting in the creation of monolayers. Previous research [23] has also demonstrated that the physical and chemical properties of the thin film formed on the silica surface, including density and thickness, are influenced not only by the functional end groups but also by other molecular components, such as PFPE segments. There are limited reports on the application of PFPE-silane as a hydrophobic agent in the field of concrete. Incorporating PFPE-silane as a core material into polymeric microcapsules to enhance the hydrophobicity of concrete holds significant value for the concrete industry.

This study employs PFPE-silane as the core material and EC as the wall material to produce hydrophobic polymeric microcapsules using a solvent evaporation method. The effects of the stirring rate and the core-to-shell ratio on the synthesized microcapsules were investigated separately by single-factor experiments. The polymeric microcapsules’ properties and chemical compositions were analyzed using scanning electron microscopy (SEM), energy dispersive X-ray spectroscopy (EDS), laser particle size analysis, thermogravimetric analysis (TG-ATG), and Fourier transform infrared spectroscopy (FTIR). Contact angle tests were conducted to examine the hydrophobic properties of the polymeric microcapsules, and the hydrophobic mechanism of PFPE-silane was investigated, providing a theoretical foundation for the preparation of hydrophobic polymeric microcapsules.

## 2. Experimental Section

### 2.1. Materials

ER (Epoxy Resin), purchased from Shanghai Autum Chemical Technology Co., Ltd. (Shanghai, China); EC powder (white granular objects, AR), purchased from Nanjing Kelong Chemical Reagent Factory (Nanjing, China); dichloromethane (DCM, AR), purchased from Chengdu Kelong Chemical Co., Ltd. (Chengdu, China); gelatin (light-yellow translucent granular objects, CP), purchased from Sinopharm Chemical Reagent Co., Ltd. (Shanghai, China); QX-18 hydrophobic agent (the main ingredient of which is PFPE-silane), purchased from Nanjing QuanXi Chemical Co., Ltd. (Nanjing, China).

### 2.2. Preparation of Microcapsules

**Figure 1:**
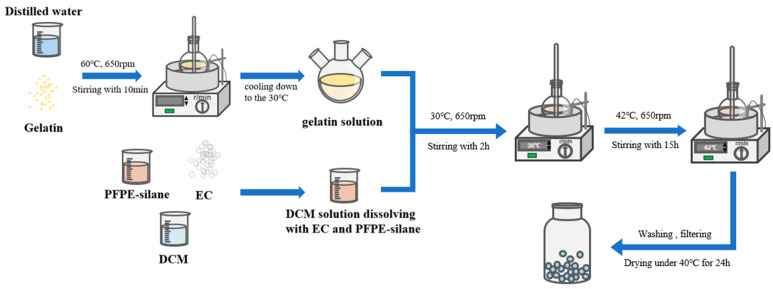
Preparation process of PFPE-silane@EC MC.

The detailed process of preparation is depicted below (Figure 1).

(1) Preparation of gelatin solution: Weighed a certain amount of gelatin (acting as emulsifier) and mixed it with distilled water. Stirred for 10 min at 60 °C and 650 rpm to fully dissolve the gelatin. Then, waited for the sol solution to slowly cool to 30 °C.

(2) Oil phase preparation: weighed a certain amount of PFPE-silane and EC, added them to DCM, stirred at room temperature at 650 r/min for 10 min, and then used ultrasonic equipment to treat the mixture, obtaining the oil phase. 

(3) Placed a three-necked flask containing gelatin solution in a water bath heating device, slowly added the oil phase to the flask, stirred for 2 h at 30 °C and 650 rpm, and fully emulsified.

(4) Raised the temperature of the water bath to 42 °C, kept the stirring rate constant, and continued stirring for 24 h. After stirring, the microcapsules were filtered out, and the excess solution on the surface of the microcapsules was cleaned with distilled water, placed in an oven, and dried at 30 °C for 24 h to obtain PFPE@EC MC.

### 2.3. Preparation of Capsule Layers

**Figure 2:**
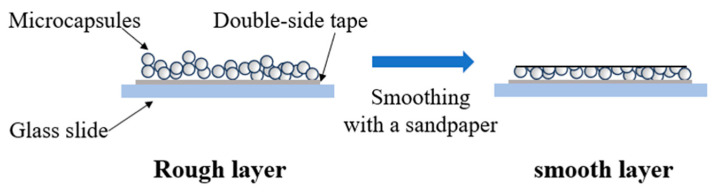
Preparation process of both rough and smooth capsule layers.

Placed the microcapsules on a glass slide to form a microcapsule layer, and repeatedly polished the microcapsule layer with 1200 Cw sandpaper to form a smooth surface. Conducted contact angle measurements to investigate the hydrophobicity of the capsule after rupture (Figure 2).

### 2.4. Characterization of Microcapsules

#### 2.4.1. Preparation Process Analysis

In order to investigate the influence of stirring rate and core-to-shell ratio on the production of microcapsules, two sets of single-factor experiments were carried out:

(1) Three sets of microcapsules were prepared at stirring rates of 500 rpm, 650 rpm, and 800 rpm, respectively, and the morphology of the prepared microcapsules was observed with an optical microscope.

(2) The microcapsules were prepared using raw materials with core-to-shell ratios (EC/PFPE-silane) of 2:1, 1:1, and 1:2, respectively, and the morphology of the prepared microcapsules was observed with an optical microscope.

#### 2.4.2. SEM-EDS Analysis 

The morphology of the PFPE-silane@EC microcapsules was characterized by using scanning electron microscopy (SEM, ZEISS Gemini 300, Carl Zeiss, Oberkochen, Germany) with an acceleration voltage of 2 kV. Energy dispersive spectroscopy (EDS, Xplore, Oxford Instruments, Oxford, UK) was used to determine the shell composition of PFPE-silane@EC microcapsules.

#### 2.4.3. Particle Size Analysis 

The particle size distribution of PFPE-silane@EC microcapsules was obtained using a laser particle size analyzer (Mastersizer 2000, Malvern Instruments Ltd., Malvern city, UK). PFPE @EC microcapsules were added to liquid cells and tested at room temperature.

#### 2.4.4. FT-IR Analysis 

In a dry environment, we took an appropriate amount of microcapsule shell material (EC), core material (PFPE-silane), and microcapsules, added the proper amount of dried KBr powder to a mortar, ground it thoroughly multiple times, and then put it into a tablet press (pressed it into a transparent thin sheet) for testing. The sample was tested using a Fourier transform infrared spectrometer (FT-IR, Thermo Scientific NicolletiS20, Thermo Fisher Scientific, Waltham, MA, USA) to determine the composition of each part of the microcapsules in the wavenumber range of 400~4000 cm^−1^.

#### 2.4.5. TG-DTG Analysis

The thermogravimetric analysis (TG) was conducted at 20~800 °C, a heating rate of 10 °C/min, and a N_2_ flow rate of 20 mL/min. The equipment used was Netzsch TG 209 F3 Tarsus (Netzsch, Selb, Germany), and synthesized microcapsules were studied using TG-DTG analysis.

#### 2.4.6. Contact Angle Measurements

The water contact angle (WCA) was measured using a drop shape analyzer (DSA30, KRÜSS Instruments Ltd., Hamburg, Germany). For the measurement, a droplet volume of 1 µL was selected. The average three-phase contact angle (θ) was measured in ten different positions of both rough and smooth layers, and the average value was recorded.

## 3. Results and Discussion

### 3.1. Optimization of Preparation Process 

#### 3.1.1. Stirring Rate

**Figure 3:**
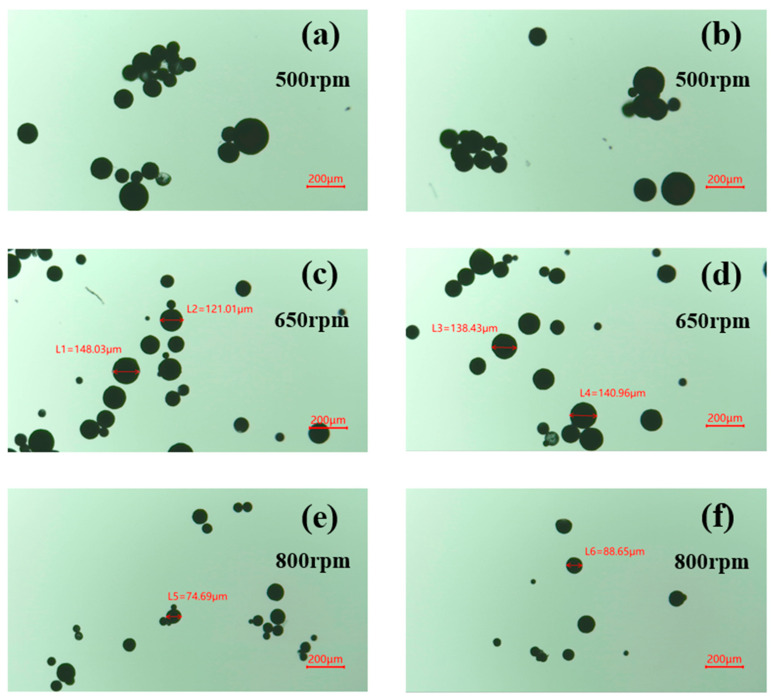
Optical microscope images of microcapsules prepared at different stirring speeds. ((**a**,**b**)—500 rpm; (**c**,**d**)—650 rpm; (**e**,**f**)—800 rpm).

Figure 3 shows the optical microscope images of microcapsules prepared at different stirring speeds. It can be seen from Figure 3 that the particle size of the synthesized microcapsules gradually decreased with an increasing stirring rate. This can be attributed to the fact that higher stirring rates promote droplet fragmentation, resulting in smaller droplet sizes and finer emulsions. Consequently, more small-diameter microcapsules were formed. At a stirring rate of 500 rpm, there was a higher aggregation of microcapsules, possibly due to inadequate dispersion caused by the lower stirring rate. At a stirring rate of 800 rpm, the microcapsules formed were small in particle size, with most of them concentrated below 100 μm. This reduction in particle size was a result of the increased turbulent kinetic energy at high stirring rates. However, microcapsules smaller than 100 μm are not suitable for application in the field of concrete. When the stirring rate was set at 650 rpm, the obtained microcapsules had a more concentrated particle size, spherical shape, and better dispersion. Both excessively high and low stirring rates are unfavorable for microcapsule production. Too low a stirring rate results in poor dispersion, while too high a stirring rate leads to excessive shear force, causing a significant decrease in yield and an excessively small average particle size that hinders normal usage. Therefore, a stirring rate of 650 rpm was used to prepare the microcapsules and for subsequent characterization.

#### 3.1.2. Core-to-Shell Ratio

**Figure 4:**
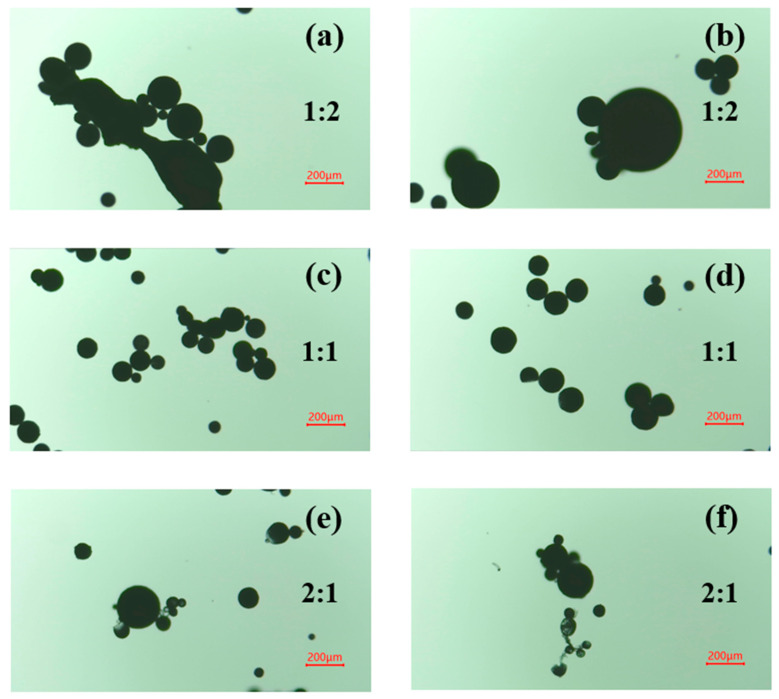
Optical microscope images of microcapsules prepared at different core-to-shell ratios. ((**a**,**b**)—1:2; (**c**,**d**)—1:1; (**e**,**f**)—2:1).

In the process of microcapsule formation, the ratio of the core to the shell has an impact on the particle size and shell thickness of the microcapsule. Generally, a larger amount of shell material results in a thicker shell layer, which requires more energy to destroy the microcapsule in practical applications. This is unfavorable for triggering the microcapsule. On the other hand, a higher core material content leads to a lower shell material content, which hinders proper encapsulation and reduces the yield of microcapsules. It is crucial to select an appropriate core-to-shell ratio. Figure 4 illustrates that a core-to-shell ratio of 1:2 results in irregular microcapsules with large particle sizes and bonding agglomeration. Conversely, a ratio of 2:1 leads to insufficient encapsulation, smaller microcapsules, and a higher occurrence of translucent and ruptured microcapsules due to thinner shell layers. However, a ratio of 1:1 produces microcapsules with better morphology. Therefore, a core-to-shell ratio of 1:1 was chosen for microcapsule preparation and subsequent characterization.

### 3.2. Morphology and Structure 

**Figure 5:**
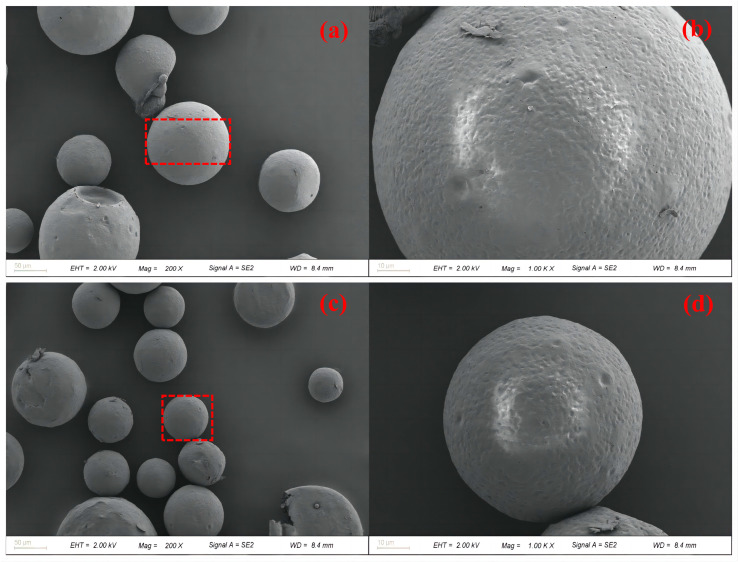
SEM images of microcapsules. ((**a**,**c**)—at 200×, (**b**,**d**)—enlarged images of red rectangular box selection area in (**a**,**c**) respectively).

**Figure 6:**
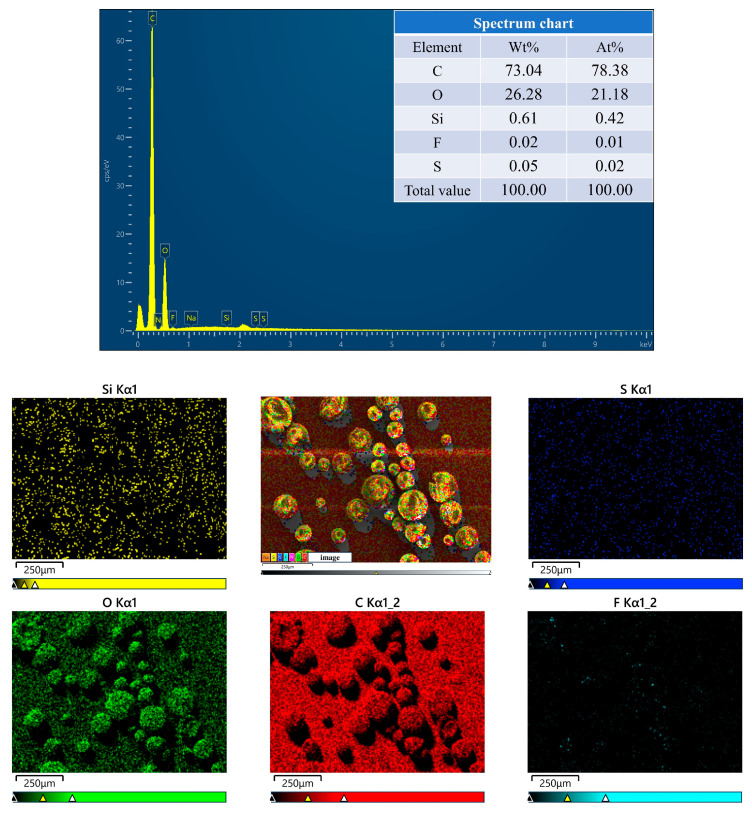
EDS images of microcapsules.

Figure 5 displays overall and partial images of PFPE-silane@EC microcapsules. The encapsulation boundary is clearly visible in Figure 5a,c, demonstrating successful encapsulation of the oil phase by EC. The microcapsules exhibit a relatively regular and dense microsphere shape with a smooth surface. However, Figure 5b,d reveal the presence of micropores on the surfaces of the microcapsules. These micropores resulted from the evaporation of the organic solvent (DCM) with a low boiling point, leading to the formation of tiny bubbles under the influence of temperature. These bubbles accumulated on the inner surface of the microcapsule shell and subsequently overflowed, leaving micropores on the surface. The presence of these micropores may potentially cause leakage of the core material, rendering the microcapsule surface hydrophobic. Figure 6 presents an EDS mapping image of the synthesized microcapsules. The surface of the microcapsules predominantly consisted of C and O chemical elements, accounting for 73.04% and 26.28%, respectively. The distribution areas of the C and O chemical elements aligned well with the microcapsule region, suggesting that the outer shell primarily comprised EC. Additionally, the image reveals the presence of Si and F chemical elements, which can be attributed to the rupture of some microcapsules, resulting in the release of PFPE-silane and its attachment to the microcapsule surface. The core material, PFPE-silane, contained a significant amount of the F chemical element, further confirming its composition.

**Figure 7:**
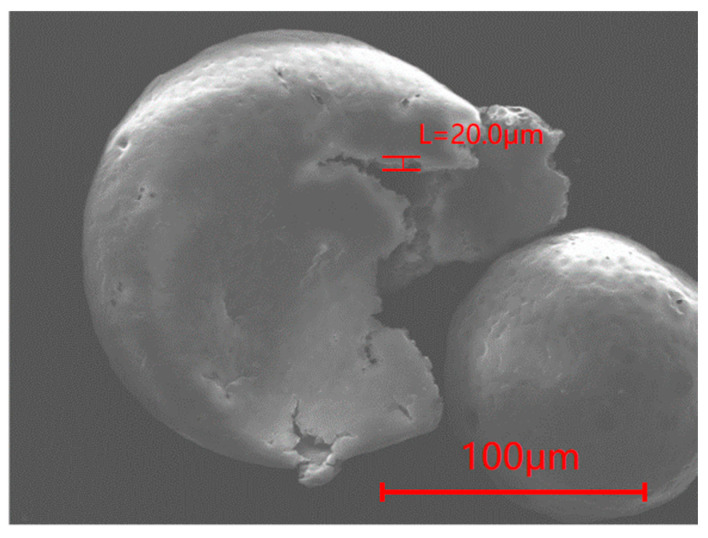
SEM images of broken microcapsules.

**Figure 8:**
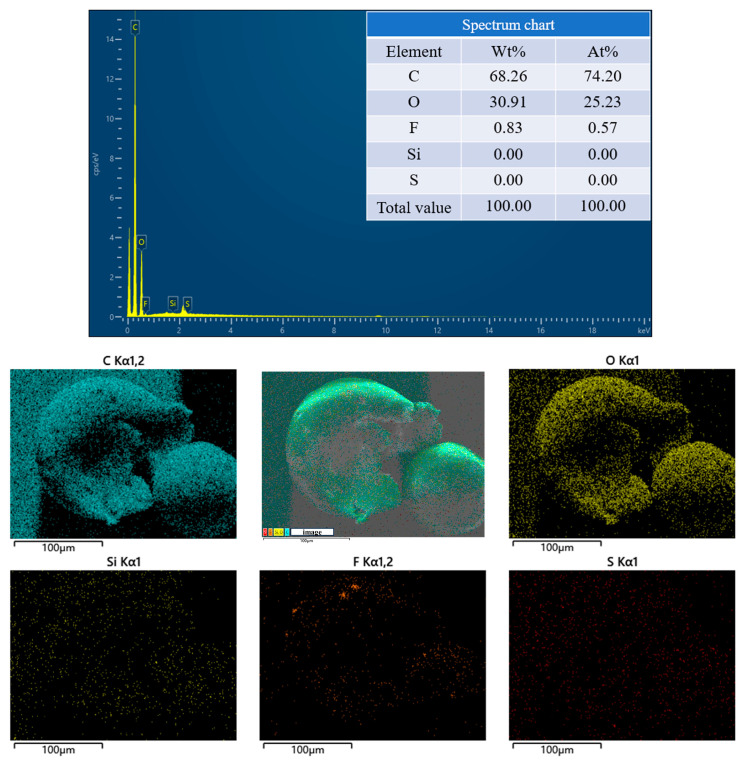
EDS images of broken microcapsules.

To investigate whether PFPE-silane was encapsulated by EC and if PFPE-silane@EC microcapsules were successfully synthesized, a grinding method was used to release the core material inside the microcapsules. The microcapsules were then analyzed by SEM-EDS. Figure 7 shows an SEM image of the microcapsules after breakage, revealing a shell layer thickness of approximately 20 μm. EDS analysis indicated that the F-element content of the microcapsules increased from 0.02% to 0.83% after breakage (Figure 8). Although the content of F-element in the microcapsules after breakage was not very high, this rise confirmed that the grinding method successfully ruptured some of the microcapsules and released part of the core material, thus verifying the successful synthesis of the microcapsules.

### 3.3. Size Distribution

**Figure 9:**
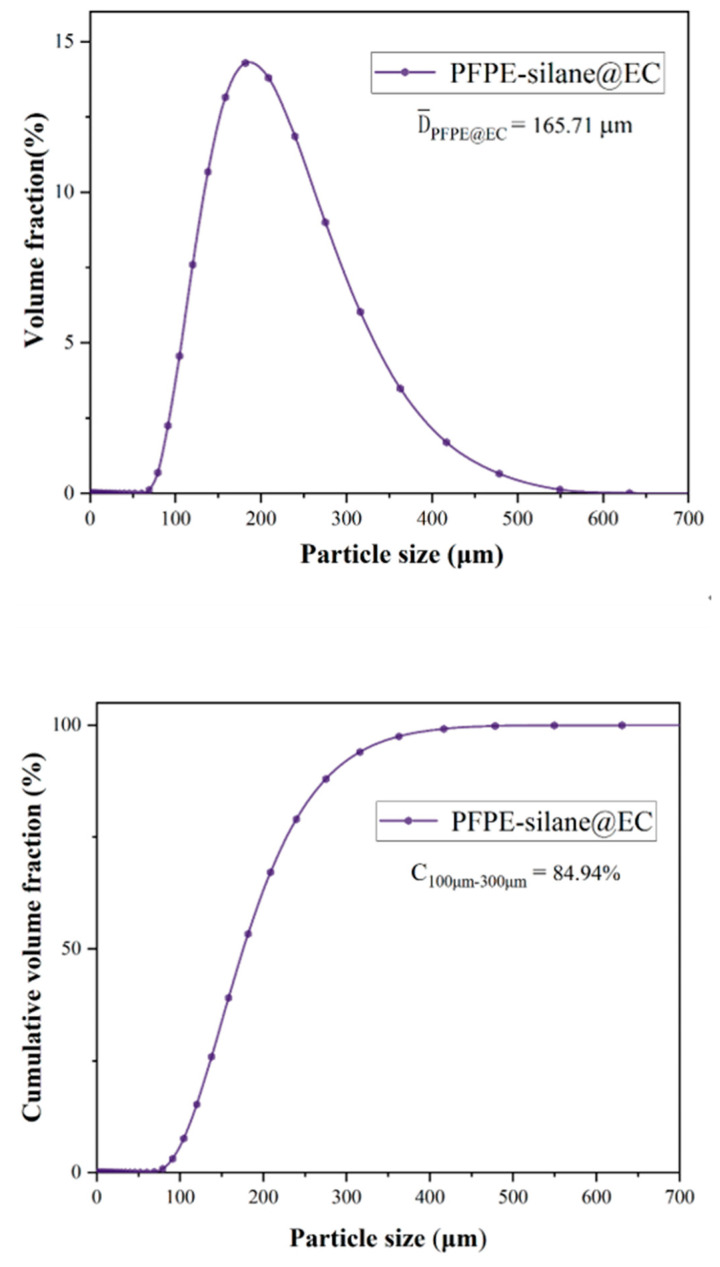
Size distribution of microcapsules.

The particle size distribution of PFPE-silane@EC microcapsules exhibited a normal distribution, with a peak at 158.49 μm. According to the literature [24,25,26], microcapsules with particle sizes ranging from 100 to 300 μm are considered most suitable for application in cement-based materials. The average particle size of the synthesized microcapsules was 165.708 μm. Figure 9 illustrates the cumulative volume fraction of different particle sizes, ranging from 100 to 300 μm, with microcapsules accounting for a high cumulative volume fraction of 84.94%. The particle size distribution of the microcapsules is predominantly concentrated between 100–300 μm. This suggests that PFPE-silane@EC microcapsules hold significant potential for application in cement-based materials. The particle size distribution of PFPE-silane@EC microcapsules is primarily concentrated between 100–300 μm, indicating small particle sizes and concentrated distribution. This observation may be attributed to the emulsification stage during the preparation process. As reported in the relevant literature, when the solvent evaporation method is employed for microcapsule preparation, higher stirring speeds during the emulsification process result in smaller average particle sizes of the capsules. This is likely due to the formation of nanoscale emulsions with increased stirring rates, leading to a narrower size distribution and smaller average particle size of the microcapsules. Therefore, maintaining an appropriate stirring rate facilitates the formation of microcapsules with a reasonable particle size distribution, enhancing their performance in cement-based materials.

### 3.4. Chemical Properties 

**Figure 10:**
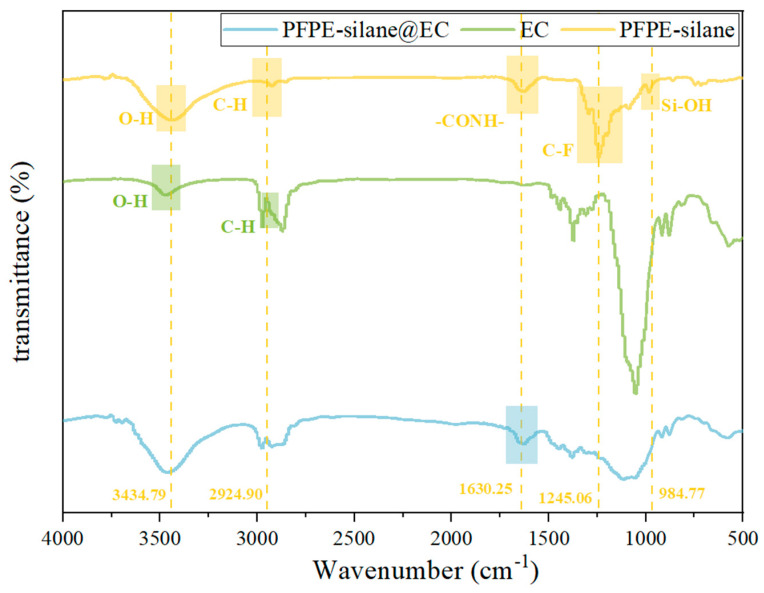
FT-IR spectrum of PFPE-silane, EC, and PFPE-silane@EC microcapsules.

Figure 10 presents the PFPE-silane’s, EC’s, and PFPE-silane@EC microcapsules’ FTIR spectra. The absorption peaks at 1245.06 cm^−1^, 1089.05 cm^−1^, and 1293.70 cm^−1^ corresponded to the stretching vibrations of -CF, -CF_2_, and -CF_3_, respectively, which confirm the presence of the PFPE component in PFPE-silane. The peaks at 2924.90 cm^−1^ and 3434.79 cm^−1^ corresponded to C-H and O-H, respectively, indicating the existence of the silane component in PFPE-silane. The peak at 984.77 cm^−1^ corresponded to the symmetric vibration of the Si-OH group [27,28]. The appearance of the Si-OH group is attributed to the hydrolysis reaction of the siloxane part in PFPE-silane, resulting in the formation of -OH and alcohol substances. Si-OH can undergo a condensation reaction with the substrate surface, forming a dense -Si-O- layer, which is crucial for the hydrophobic properties of PFPE-silane. In Figure 10, it is observed that the characteristic C-F and O-H peaks of PFPE-silane are not clearly visible in the microcapsule spectrum. This could be attributed to the presence of a prominent peak at 1052.02 cm^−1^ in the EC spectrum, which might have interfered with the characteristic peaks of the PFPE-silane. The characteristic peak at 1630.25 cm^−1^ corresponded to -CONH-, a spacer group in the molecular structure of the PFPE-silane used to connect the PFPE part to the silane part. The FTIR spectrum of the PFPE-silane@EC microcapsules also exhibited corresponding peaks, confirming the successful encapsulation of PFPE-silane in the microcapsule shell. In the FTIR spectrum of EC, peaks at 2974.58 cm^−1^ and 3467.81 cm^−1^ were observed, corresponding to C-H and O-H, respectively [29,30]. These peaks were also present in the spectrum of PFPE-silane@EC microcapsules, indicating the successful synthesis of PFPE-silane and EC in the PFPE-silane@EC microcapsules.

### 3.5. Thermal Stability

**Figure 11:**
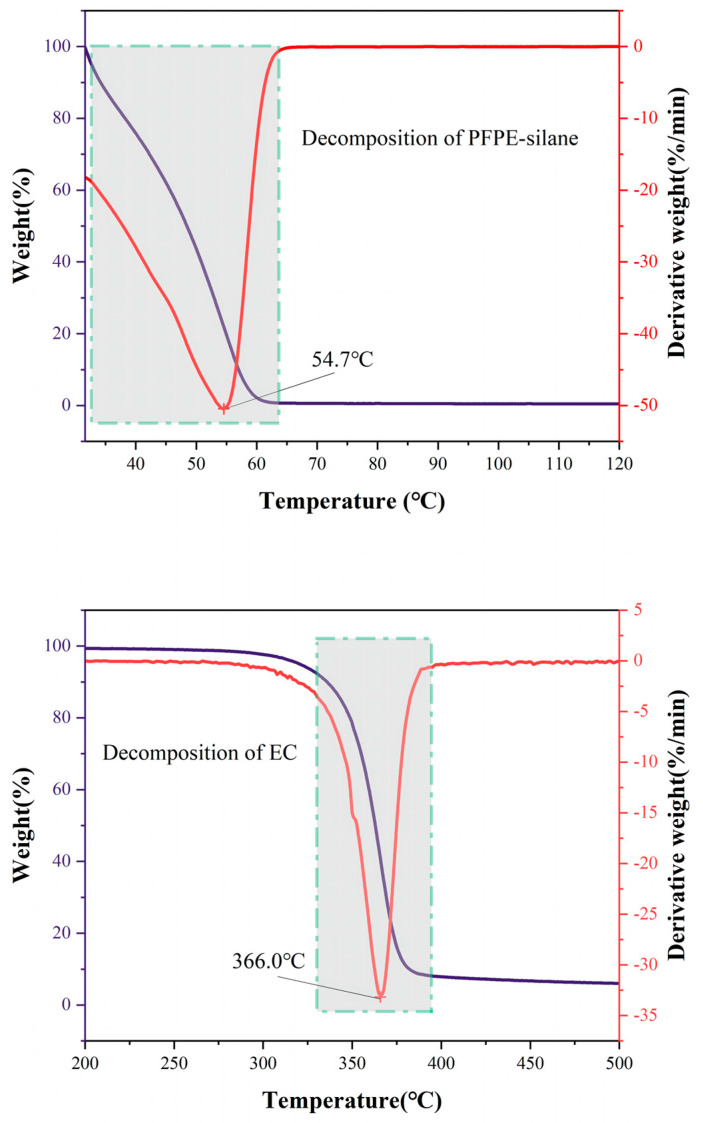
TG−DTG curves of PFPE-silane and EC.

**Figure 12:**
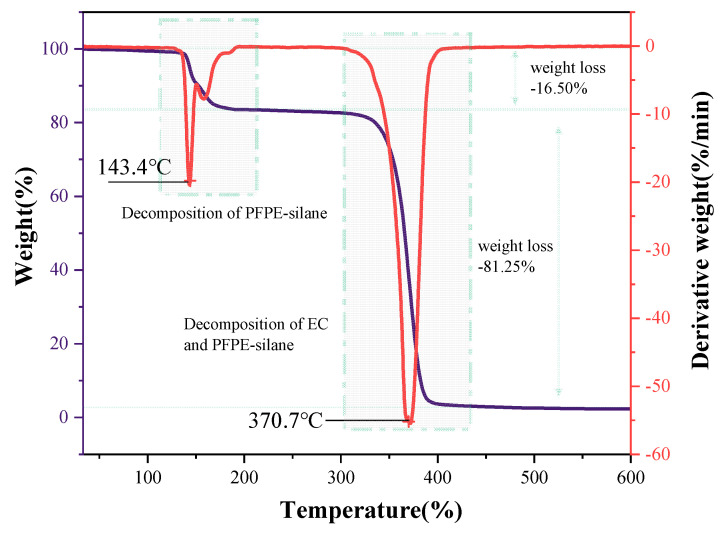
TG−DTG curve of PFPE-silane@EC microcapsules.

The thermal properties of the shell material, core material, and overall microcapsule were measured using the TG-DTG method. The weight of the sample gradually decreased with the increase in temperature, as observed in Figure 11 and Figure 12. Figure 11 shows that the decomposition temperature of the microcapsule’s shell material (EC) was 366 °C, while the decomposition temperature of the core material (PFPE-silane) was 54.7 °C. This indicates that PFPE-silane decomposed earlier than EC, which also occurred during the decomposition process of the microcapsule. The lower decomposition temperature of PFPE-silane compared to EC made it susceptible to decomposition due to the heat generated by the cement hydration reaction, which is unfavorable for its application in cement. However, the thermogravimetric curve of the microcapsules (Figure 12) shows that the first weight loss peak was delayed to 143.4 °C due to the protection provided by the shell material (EC). At this temperature, the micropores on the surface of the microcapsules released PFPE-silane, and some decomposition of PFPE-silane occurred. It is worth noting that there were two peaks around 143.4 °C, which may be due to PFPE-silane decomposition. PFPE-silane is a polymer compound with long chains in its molecule. It is speculated that an increase in temperature may lead to the decomposition of PFPE-silane within the shell, and the evaporation of these decomposition products could cause the formation of different peaks. The temperature of 143.4 °C is higher than the temperature during the cement hydration process (80 °C), ensuring the protection of PFPE-silane and preventing premature failure caused by hydration heat release. This improved stability allows the microcapsules to have potential applications in cement-based materials.

The thermal decomposition process of synthesized microcapsules can be divided into three stages. Firstly, before reaching 139.5 °C, there was a slight weight loss of only 0.04%. This is due to the evaporation of residual liquid on the microcapsule surface, resulting in a gradual decrease in the curve. Secondly, between 139.5 °C and 354.5 °C, there was a significant decrease in the thermogravimetric curve, with a weight loss of approximately 16.5% and a peak derivative weight of 17.86%/min. The size of the pores on the surface of the capsule was around 20 μm in width and the shell layer had curved pores in the cross-section, which is not conducive to PFPE leakage at low temperatures. When the temperature rose to a certain level, however, the expansion and deformation of micropores on the microcapsule shell allowed the release of PFPE-silane, causing the decomposition of the unprotected core material and the release of heat. Lastly, at temperatures ranging from 354.5 °C to 800 °C, there was a substantial weight loss of 81.25%, and the peak derivative weight reached 55.07%/min. This occurred because the decomposition temperature of EC was reached, leading to the oxidation and decomposition of the anhydrous-glucose polymeric chains in EC [31,32], resulting in heat release. It is worth noting that there was a 4 °C difference between the peak for pure EC (366 °C) and the peak for the EC shell of the microcapsule (370.7 °C). This difference can be attributed to the slight variations in the microcapsule shell. As the superhydrophobicity component of the core material, the hydroxyl groups on the PFPE-silane may attach to the surface of the shell layer, introducing variations in the microcapsule shell compared to that of pure ethylcellulose. The second thermal decomposition process of the microcapsule exhibited a much higher peak derivative weight (55.07%/min) than the EC thermal decomposition process (32.80%/min). This difference is attributed to the partial decomposition of PFPE-silane in the first thermal decomposition process of the microcapsule. Following the rupture of EC, the remaining PFPE-silane decomposed and released heat along with EC.

### 3.6. Hydrophobicity of Microcapsules in Layers 

**Figure 13:**
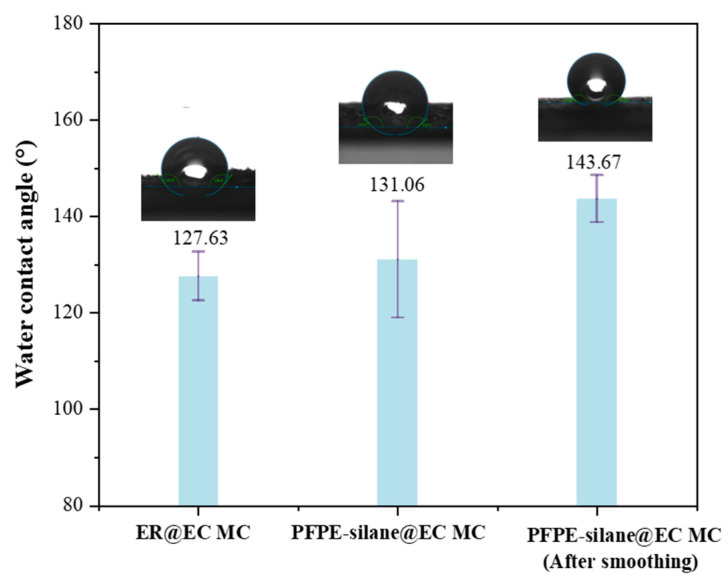
Water contact angles of different microcapsule layers.

Contact angle measurement is a widely used method for studying the interaction between solids and liquids at interfaces, particularly for assessing wettability. The contact angle (denoted by θ) is determined by the relative molecular forces within the liquid and between the liquid and the solid. A large contact angle indicates strong liquid cohesion and weak solid–liquid adhesion. When the contact angle exceeds 90°, the material is generally considered hydrophobic, and a contact angle greater than 150° indicates superhydrophobic properties. In this study, we prepared ER@EC microcapsules and PFPE-silane@EC microcapsules using the same method. These different capsules were attached to a glass slide to form a microcapsule layer, which was then smoothed with sandpaper. The results revealed that the contact angles of ER@EC MC and PFPE-silane@EC MC were 127.63° and 131.06°, respectively, fulfilling the criterion for hydrophobic materials (90°) (Figure 13). The hydrophobicity of the capsule layers of ER@EC MC and PFPE-silane@EC MC was primarily attributed to the rough structure formed by micrometer-sized particles and the inherent hydrophobicity of EC itself. EC exhibits high hydrophobicity due to the presence of ethyl groups (CH_3_CH_2_-) in its molecule, which are non-polar hydrophobic groups. The contact angle of the unsmoothed PFPE-silane@EC microcapsule layer (131.06°) was larger than that of the ER@EC microcapsule layer (127.63°). This difference could be attributed to the presence of numerous micropores in the PFPE-silane@EC shell, resulting in the leakage of the hydrophobic core material (PFPE-silane), which subsequently covered the surface of the microcapsules and enhanced their hydrophobicity. After smoothing, the contact angle of the PFPE-silane@EC microcapsule layer increased by 9.62% compared to that of the unsmoothed layer, representing a significant improvement. Based on Section 3.2, the increase in contact angle can be attributed to two reasons. Firstly, the contact angle increased because the microcapsule layer became smoother after being polished with sandpaper. Secondly, the contact angle increased because the destruction of the external force led to the release of the core material inside the microcapsule, forming a hydrophobic layer on the surface of the microcapsules. These findings highlight the excellent hydrophobic properties of PFPE-silane.

**Figure 14:**
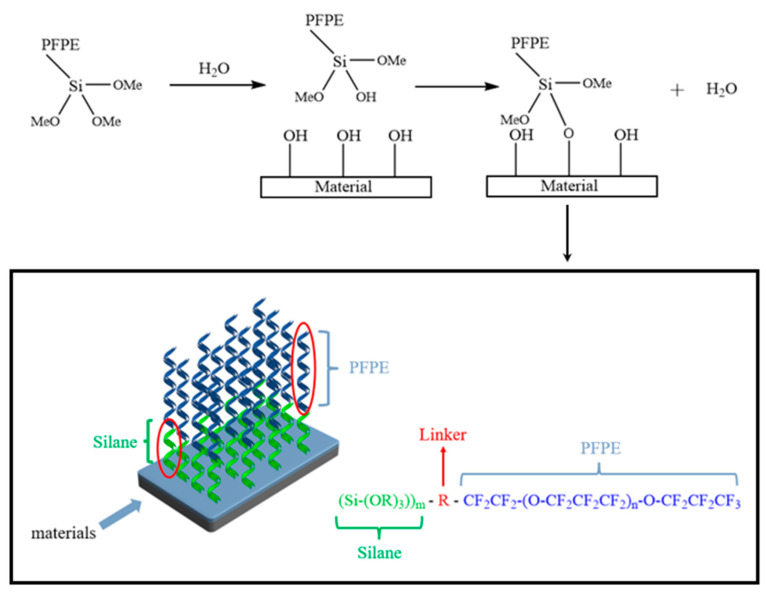
PFPE-silane hydrophobicity mechanism.

The excellent hydrophobic performance of PFPE-silane is attributed to the modification of perfluoropolyether with siloxane, which allows it to possess both perfluoropolyether and siloxane properties (Figure 14). PFPE and siloxane have different hydrophilicity and lipophilicity, and each plays a distinct role in forming hydrophobic films. Koji Honda et al. [23] discovered that the hydrophobicity of the compound improves with longer and more regular fluorinated chain segments. Similarly, the material’s hydrophobicity is enhanced with longer PFPE chain segments. During the single-molecule assembly process, PFPE segments migrate towards the surface of the membrane layer, aligning outward, while the siloxane portion tends to approach the base material. The end of the siloxane undergoes a hydrolysis reaction, forming -OH, followed by a condensation reaction and self-condensation reaction with the base material. This forms more -Si-O- bonds and results in a strong adhesion with the material substrate. The single-molecule assembly process allows PFPE-silane to create a double-layer single-molecule coating on the material surface, exhibiting excellent hydrophobic properties.

## 4. Conclusions

In this study, a simple and cost-effective preparation method was used to create PFPE-silane@EC polymeric microcapsules. PFPE-silane was used as the core material, and EC was used as the wall material. The composition of the polymeric microcapsules was analyzed using SEM, EDS, FTIR, and other testing methods. The following conclusions were drawn:

(1) In the process of preparing microcapsules, the stirring rate has an impact on the particle size and distribution of the synthesized microcapsules. Additionally, the core-to-shell ratio affects the morphology of the microcapsules and the thickness of the shell layer. By using a stirring rate of 600 rpm and a core-to-shell ratio of 1:1, it is possible to prepare microcapsules with uniform particle size distribution and desirable morphology.

(2) The PFPE-silane@EC polymeric microcapsules had a circular shape with a high balling rate. The thickness of the shell layer of the microcapsules was about 20 μm. The surfaces of the microcapsules had micropores, which were formed during the preparation process due to solvent evaporation. The EC wall of the polymeric microcapsules had good permeability, allowing the release of core materials and making the surface hydrophobic.

(3) The average particle size of the PFPE-silane@EC polymeric microcapsules was 165.71 μm, and the cumulative volume fraction between 100 and 300 μm was 84.94%, making them suitable for application in the cement field.

(4) PFPE-silane has a low decomposition temperature, which is lower than the temperature of cement hydration heat release. However, when used as the core material in polymeric microcapsules, the high decomposition temperature of the EC wall material can protect the core material and raise its decomposition temperature from 54.7 °C to 143.4 °C. This allows PFPE-silane to avoid thermal decomposition during cement hydration and effectively exhibit its hydrophobic properties. 

(5) The combination of PFPE segments and silane in PFPE-silane maximized its hydrophobic properties, resulting in a water contact angle of 143.67°.

## Data Availability

The data presented in this study are available on request either from the first or corresponding authors.
